# Supplementary material for: Dipotassium Glycyrrhizinate on Melanoma Cell Line: Inhibition of Cerebral Metastases Formation by Targeting NF-kB Genes-Mediating MicroRNA-4443 and MicroRNA-3620—Dipotassium Glycyrrhizinate Effect on Melanoma
Source: Int J Mol Sci. 2022 Jun 29;23(13):7251. doi: 10.3390/ijms23137251 (PMC9266887; doi:10.3390/ijms23137251)
Supplement: Supplementary file 1 [file ijms-23-07251-s001.zip › ijms-1720266-supplementary.pdf]

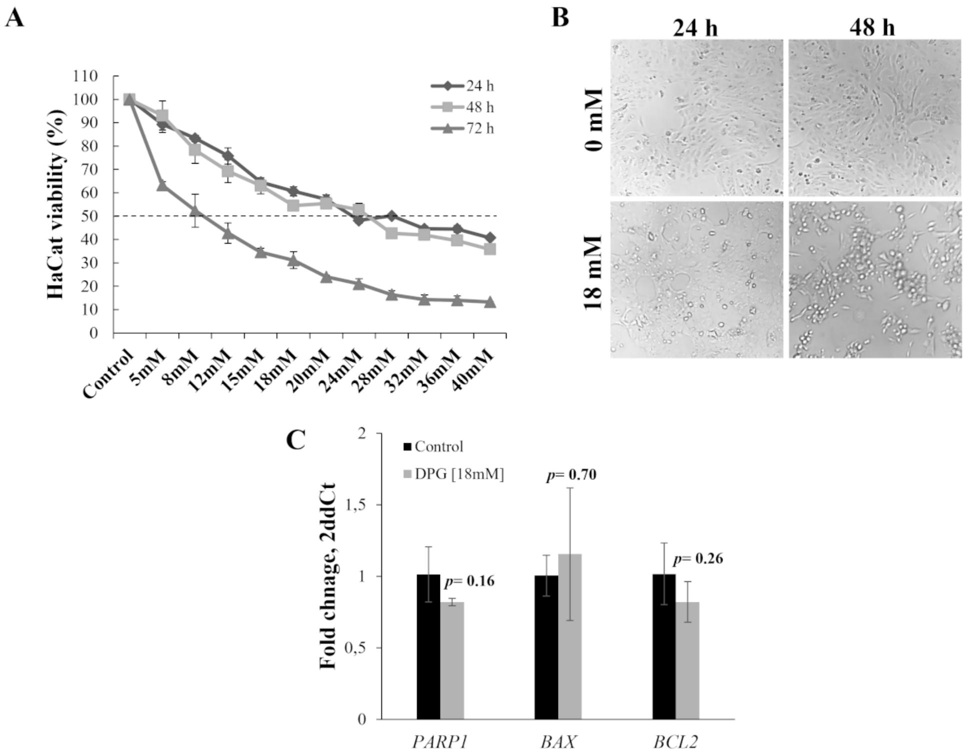

Supplementary Figure S1. (A) Dipotassium glycyrrhizinate (DPG) inhibits cell viability of HaCat cells treated with different DPG concentrations for 48 and 72h by MTT assay, and reduction in cell viability was observed only with high DPG concentrations (24-40mM) for 48h or lower DPG concentrations (8-40mM) for 72h. **(B)** HaCat nuclear morphological change was observed 48h after DPG. **(C)** Normal Poly [ADP-ribose] Polymerase 1 (*PARP-1*) (*p-value* = 0.16), *BAX* (*p-value* = 0.70), and *BCL-2* (*p-value* = 0.26) mRNA expression level after 48h of DPG treatment, compared to control cells. For all assays, data represent means and standard deviations of representative experiments performed in triplicate. Statistics were performed in a two-tailed T-test with an alpha error of 0.05.
